# Assessment of Fat distribution and Bone quality with Trabecular Bone Score (TBS) in Healthy Chinese Men

**DOI:** 10.1038/srep24935

**Published:** 2016-04-26

**Authors:** Shan Lv, Aisen Zhang, Wenjuan Di, Yunlu Sheng, Peng Cheng, Hanmei Qi, Juan Liu, Jing Yu, Guoxian Ding, Jinmei Cai, Bin Lai

**Affiliations:** 1 Department of Gerontology, the First Affiliated Hospital with Nanjing Medical University, 300 Guangzhou Road, Nanjing 210029 Jiangsu, P.R. China

## Abstract

Whether fat is beneficial or detrimental to bones is still controversial, which may be due to inequivalence of the fat mass. Our objective is to define the effect of body fat and its distribution on bone quality in healthy Chinese men. A total of 228 men, aged from 38 to 89 years, were recruited. BMD, trabecular bone score (TBS), and body fat distribution were measured by dual-energy X-ray absorptiometry. Subcutaneous and visceral fat were assessed by MRI. In the Pearson correlation analysis, lumbar spine BMD exhibited positive associations with total and all regional fat depots, regardless of the fat distribution. However, the correlation disappeared with adjusted covariables of age, BMI, HDL-C, and HbA1c%. TBS was negatively correlated with fat mass. In multiple linear regression models, android fat (and not gynoid, trunk, or limbs fat) showed significant inverse association with TBS (β = −0.611, P < 0.001). Furthermore, visceral fat was described as a pathogenic fat harmful to TBS, even after adjusting for age and BMI (β = −0.280, P = 0.017). Our findings suggested that body fat mass, especially android fat and visceral fat, may have negative effects on bone microstructure; whereas body fat mass contributes to BMD through mechanical loading.

A number of studies have been carried out to investigate the relationship between fat mass and bones. Fat has been proposed to exert a harmful role in the development of osteoporosis by producing inflammatory cytokines and imparting insulin resistance^1^,^2^. On the contrary, excess fat increases mechanical loading on the bone and links to higher bone mineral density (BMD, g/cm^2^)^3^,^4^. Therefore, the exact relationship between fat and bones is still unknown.

In order to address the controversial results, it is important to consider that adipose tissues are extremely active metabolically; however, not all adipose tissues are metabolically equivalent^5^. In the early 1990 s, Heiss *et al.* reported an association between body fat distribution and BMD, with the android distribution presenting a higher BMD^6^. In addition, a research on healthy women showed that the visceral and subcutaneous fat have opposite effects on the skeleton^7^. In a latest study involving postmenopausal Korean women, it was observed that a relatively large visceral fat and small subcutaneous fat may have a detrimental effect on bone quality^8^.

Bone quality and bone quantity are two vital components of bone strength, but BMD suffers from the lack of evaluation on the bone quality^9^,^10^. In other words, there are factors other than the bone mass, which influence bone strength and fracture risk, including microarchitectural deterioration of bone. Thus, BMD alone cannot explain the inconsistency relationship between fat and bone metabolism. The trabecular bone score (TBS) is a new grey-level texture parameter that can be computed from DXA images and makes up for the defects of the BMD^11^. Previous studies have indicated that type 2 diabetes is associated with increased fracture risk; but diabetic patients show higher BMD compared with nondiabetic individuals^12^,^13^. An explanation to this problem was provided by a recent clinical study, which revealed that TBS predicts osteoporotic fractures in patients with diabetes^14^. Furthermore, Kolta *et al.* found that in postmenopausal women, lumbar osteoarthritis leads to an increase in lumbar spine BMD, while TBS is not affected by lumbar osteoarthritis^15^. Although TBS is not a diagnostic tool for osteoporosis, several studies have shown that it can be an effective addition to enhance fracture risk prediction^16^,^17^,^18^.

Different types of body fat may exert distinct effects on bones and only a few studies have used TBS as an evaluation index in this area^8^,^19^,^20^. Estrogen deficiency is a known cause of low BMD, as it increases adipocyte differentiation^21^. Therefore, in the present study, we chose healthy men who did not suffer from metabolism disorders, and were not on medications, such as glucocorticoid, estrogen, and bisphosphonate. Herein, we aimed to better understand the characteristics between TBS and body fat distribution in comparison to BMD.

## Methods

### Study setting and participants

The clinical study was approved by the Ethics Committee of the First Affiliated Hospital of Nanjing Medical University, Jiangsu, China, in accordance with the Declaration of Helsinki. Written informed consents were obtained from all participants.

The inclusion criteria for the subjects included normal levels of plasma cortisol, calcium, phosphorus, FT3, FT4, TSH, and fasting glucose (less than 126 mg/dl). However, patients with tumor, significant liver disease, creatinine clearance of <30 ml/min, rheumatoid arthritis, previous pathological fractures, and patients taking the medicines that could affect bone mass (such as bisphosphonate, calcitonin, estrogens, Vitamin D, glucocorticoids) were excluded.

Ultimately, 228 healthy Chinese men, aged 38–89 years, were selected for this study. Fasting levels of glycated hemoglobin, cholesterol, triglycerides, low-density lipoprotein, and high-density lipoprotein were obtained.

### Measurement of BMD

DXA scans were performed and analyzed according to the manufacturer’s instructions, at the First Affiliated Hospital of Nanjing Medical University. Bone mineral density (BMD) measurements were recorded for the lumbar spine from L1 through L4 (L1–L4) and for the femoral neck (and total hip). All scans were reprocessed centrally using the same software (Hologic Discovery W). BMD values were determined by automated analysis, which were altered by the technologist, if necessary. No magnification effects were reported for the densitometer employed in this study. The instruments used, exhibited stable long-term performance (coefficient of variation (CV) < 0.5%) and satisfactory *in vivo* precision.

### Measurement of TBS

All trabecular bone score (TBS) measurements were performed at the First Affiliated Hospital of Nanjing Medical University using TBS iNsight® software (Version 2.0.0.1, Med-Imaps, Bordeaux, France). Each of the lumbar spine raw DXA images was uploaded into the TBS iNsight software. Lumbar spine TBS was then evaluated using the patented algorithm in the same regions of measurement as those used for the lumbar spine BMD (mask of the region of interest and edge detection were copied from the DXA scans), with lumbar spine TBS calculated as the mean value of the individual measurements for vertebrae L1–L4.

### Measurement of body fat distribution

Total and regional (trunk, android, gynoid, limbs) fat masses were also measured by DXA and analyzed by Encore Software 11. Trunk fat was designated from the pelvis cut (lower boundary) to the neck cut (upper boundary)^5^. Android fat was defined from the pelvis cut to above the pelvis cut by 20% of the distance between the pelvis and neck cuts. Gynoid fat was described from the lower boundary of the umbilicus to a line equal to twice the height of the android fat distribution.

### Measurement of SAT and VAT

An abdominal Magnetic Resonance Imaging (MRI) was performed on 95 men in the fasting state and included measurements of subcutaneous adipose tissue (SAT) and visceral adipose tissue (VAT) at the lumbar 4–5 level using a 3.0 T MRI system (MAGNETOM trio, Siemens, Germany) with a phased-array surface coil. Areas of SAT and VAT were estimated on T1-weighted sequences using a validated software^22^,^23^.

### Statistical analysis

Descriptive data for the subject characteristics were presented as mean ± standard deviation (S.D.) or n. The association of TBS or BMD with clinical characteristics and body composition was determined using Pearson correlation analysis. We performed one-way ANOVA and post hoc analysis by Tukey’s correction to analyze TBS, lumbar BMD, total and android fat mass among normal weight, overweight, and obesity groups, according to their BMI (18.5–23.9 kg/m^2^, 24–28 kg/m^2^,  ≥28 kg/m^2^, respectively). We further applied multiple linear regression models for TBS and BMD analyses, using age, BMI, HDL-C, HbAc1%, and different regional fat mass data. All statistical analyses were performed using IBM SPSS Statistics for Windows (Version 20, IBM Corp, Armonk, NY, USA) and P < 0.05 was considered statistically significant.

## Results

**Figure 1:**
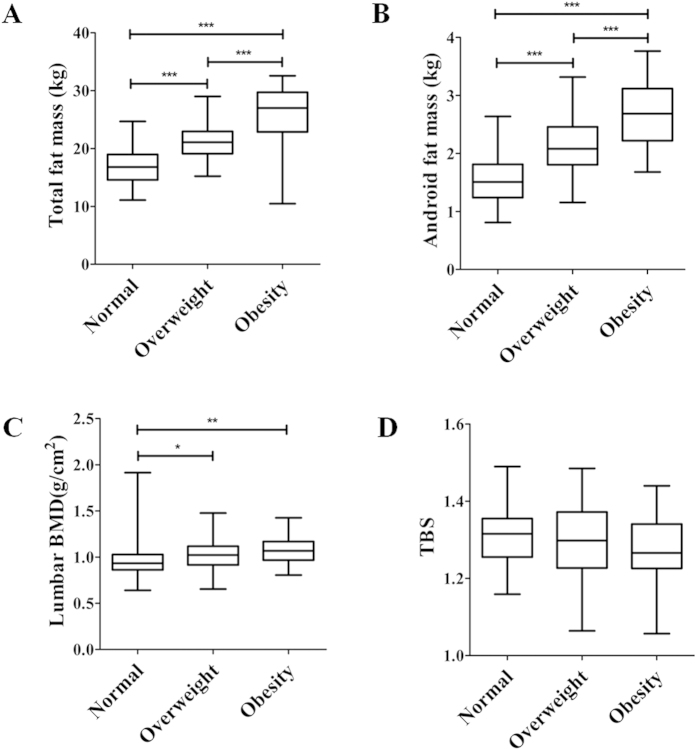
Total fat mass (**A**), Android fat mass (**B**), BMD (**C**), and TBS (**D**) of the lumbar spine in normal weight, overweight, and obesity men. One-way ANOVA was used among the three groups according to their BMI, and post hoc analysis was performed by Tukey’s correction. BMI for normal: 18.5–23.9 kg/m^2^; overweight: 24–28 kg/m^2^; obesity: ≥28 kg/m^2^. *P < 0.05; **P < 0.01; ***P < 0.001.

A total of 228 healthy Chinese men were included in the analysis, with data obtained from the baseline visit of a clinical trial (Table 1). Of these 228 participants, 78 had a lower BMI of 24 kg/m^2^ (normal), 111 participants showed a BMI between 24 and 28 kg/m^2^ (overweight), and the remaining 39 had a BMI ≥ 28 kg/m^2^ (obese)^24^. As expected, total body fat mass gradually increased with the weight gain; overweight subjects had greater total body fat than normal (P < 0.001), while obese participants had greater total body fat than the overweight group (P < 0.001) (Fig. 1A). Similar results were observed for android fat mass (Fig. 1B). Interestingly, lumbar spine BMD increased with higher BMI (Fig. 1C), but there was no statistical association between TBS and BMI among the three groups (Fig. 1D).

**Figure 2:**
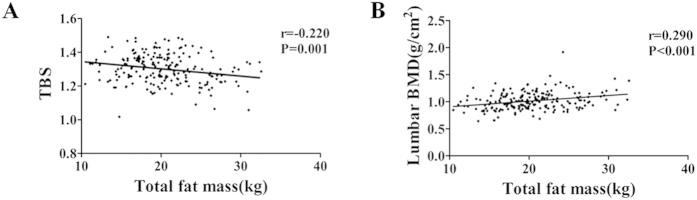
Pearson’s correlation between Total fat mass and Lumbar spine TBS or BMD. (**A**) Total fat mass correction with Lumbar spine TBS. (**B**) Total fat mass correction with Lumbar spine BMD.

We further carried out the Pearson correlation analysis, and found that the whole body fat mass correlated significantly with the lumbar spine BMD (r = 0.290, P < 0.001). In contrast, there was a negative correlation between TBS and whole body fat (r = −0.220, P = 0.001) (Fig. 2).

**Figure 3:**
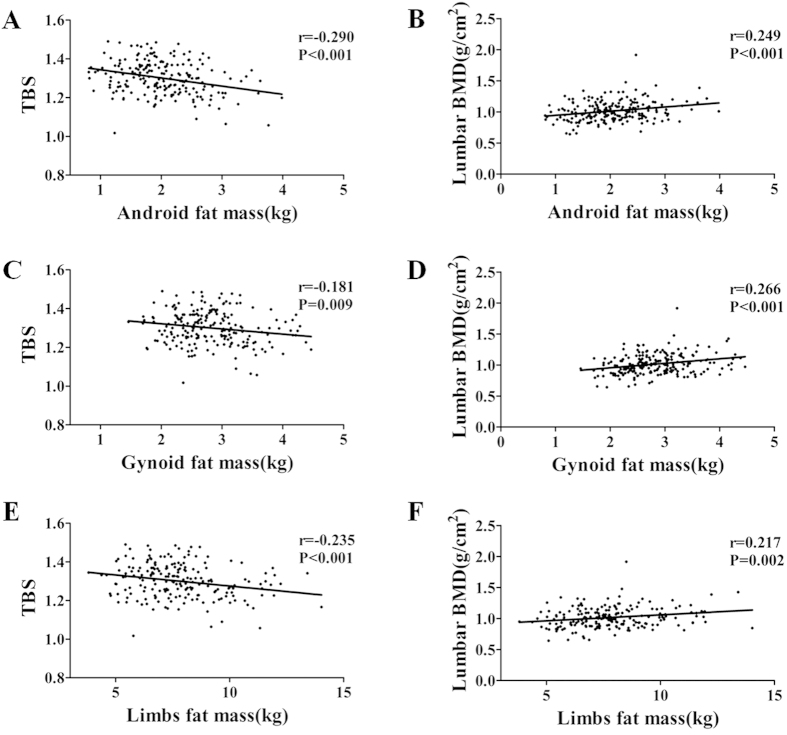
Pearson’s correlation between regional adiposity parameters and Lumbar spine TBS or BMD. (**A**,**B**) Android fat mass correction with Lumbar spine TBS or BMD. (**C**,**D**) Gynoid fat mass correction with Lumbar spine TBS or BMD. (**E**,**F**) Limbs fat mass correction with Lumbar spine TBS or BMD.

The linear negative correlation between android fat and TBS at the lumbar spine (r = −0.290) was statistically significant at P < 0.001(Fig. 3A). Further analysis revealed a greater influence of android fat on TBS than gynoid fat (r = −0.181) or four limbs fat (r = −0.235) (Fig. 3C,E). However, lumbar spine BMD showed a positive relationship with android, gynoid, and limbs fat mass, in contrast to TBS (Fig. 3B,D,F). In the multiple linear regression models (Table 2), android fat was inversely associated with TBS (β = −0.611, P < 0.001), whereas gynoid and limbs fat did not show any association with TBS. Additional data on the correlation of adjusted covariables, such as age, BMI, HDL-C, and HbA1c% have been presented in Table 2. The lumbar spine BMD was not found to be related with fat mass and fat distribution.

**Figure 4:**
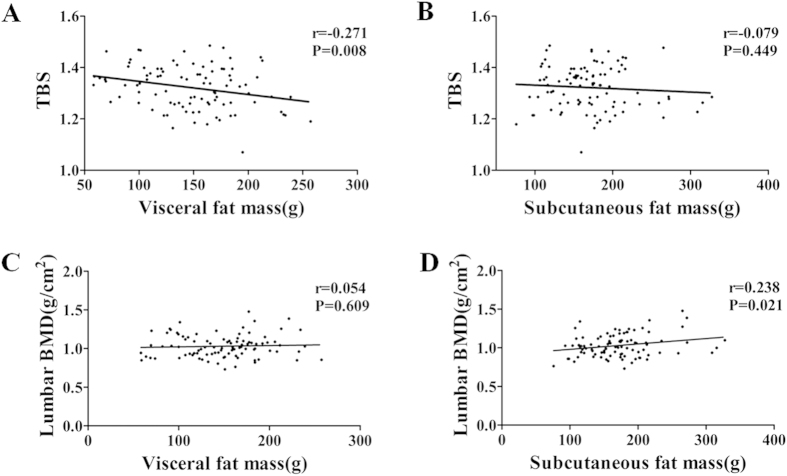
Pearson’s correlation between Visceral or Subcutaneous fat mass and Lumbar spine TBS or BMD. (**A,C**) Visceral fat mass correction with Lumbar spine TBS or BMD. (**B**,**D**) Subcutaneous fat mass correction with Lumbar spine TBS or BMD.

Subgroup analyses by MRI suggested that lumbar spine TBS was negatively correlated with the visceral fat (r = −0.271, P = 0.008), but not with subcutaneous fat (r = −0.079, P = 0.449) (Fig. 4A,B). However, contrasting results were obtained for BMD, which showed a significant positive correlation with subcutaneous fat (r = 0.238, P = 0.021), but not with visceral fat (r = 0.054, P = 0.609) (Fig. 4C,D). On the other hand, multiple linear regression model analysis showed that visceral fat mass was inversely associated with TBS (β = −0.280, P = 0.017) with adjusted covariables of age and BMI. Nevertheless, in this analysis, the correlation between BMD and subcutaneous fat disappeared (Table 3).

Analysis of data from clinical trials provided additional information on the indicator of TBS in healthy Chinese men. Lumbar spine TBS showed a positive correlation with HDL-C (r = 0.161, P = 0.015) (Table 1). In the multiple linear regression analysis (Table 2), however, this correlation disappeared, and the HDL-C showed a weak correlation with lumbar spine BMD (β = 0.151, P = 0.034).

## Discussion

Our data demonstrated that BMD, but not TBS, is associated with higher body mass index (BMI), which is consistent with previous observations^25^. Furthermore, we found that whole body fat, android fat, gynoid fat, and limbs fat mass, regardless of fat distribution were all positively related to BMD in healthy Chinese men. However, in the multiple linear regression models, android fat and gynoid fat, or subcutaneous fat and visceral fat were not correlated with BMD, even after adjusting for confounding factors. This discrepancy may have been caused by collinearity of BMI with weight related parameters. Although lumbar spine TBS was derived from a DXA image, its relationship with BMI was very weak as compared with BMD. As an important part of body weight, greater fat mass may increase mechanical loading on the bones, which links to higher BMD^4^. Some researchers have used multiple regression analysis in an attempt to gain further insight into the relationship between fat and bones. Nevertheless, body weight and fat mass are very closely interrelated and do not meet the accepted criteria for independent variables^26^. Moreover, BMD may be confused by bone size since BMD measurement by DXA is a 2-dimensional reflection of the 3-dimensional structures^27^. Therefore, BMD used in osteoporosis diagnosis has intrinsic defects.

Additionally, BMD is only an assessment of bone quantity and does not provide information on bone quality. TBS, which was derived from DXA images, is related to the structural condition of bone microarchitecture and serves as a better indicator for the evaluation of bone quality. Several studies have shown that low TBS is an important osteoporosis fracture risk factor^16^,^17^,^18^, is responsive to treatment, and plays a role in secondary osteoporosis^11^. In our study, TBS was found to be negatively correlated with total body fat, which provides an explanation for obesity associated fracture risk.

Compared with whole body fat mass, android fat mass has been suggested to be a better indicator of obesity status for an individual, because android fat is strongly associated with increased risks of hypertension, cardiovascular disease, insulin resistance, as well as type 2 diabetes^28^, and influences potential health parameters the most. In this study, whole body fat was divided into several types; and android fat was observed to have the greatest influence on TBS even after adjusting for confounding factors. Therefore, we firmly believe that more android fat is associated with greater fracture risk, independent of BMD. Measurement of android fat weight is more sensitive in bone microarchitecture assessment and different fat distributions have different effects on bone quality.

An issue that requires special consideration in studies related to relationships between bones and fat is the effect of hormonal or metabolic factors on fat and fat distribution. Android fat, especially VAT, express higher levels of inflammatory factors (including interlukin-6 (IL-6) and TNF-α) in obese individuals than in lean individuals^29^. These inflammatory cytokines are mediators of osteoclast differentiation and bone resorption^30^. In addition, there is significant evidence of VAT imparting a greater risk of insulin resistance and hyperlipidemia than SAT^27^,^31^. Hence, it is also possible that VAT and SAT differ in their impact on bones. Of interest, subgroup analysis by MRI suggested that lumbar spine TBS was negatively associated with visceral fat, but not subcutaneous fat. Recently, this negative relationship between android fat and TBS was attributed to the greater diffraction of X-rays by thick soft tissues^15^. However, in the present study, SAT and VAT of android fat mass expressed diverse associations with TBS and BMD; thereby rendering this explanation inadequate in analyzing the relationship between bone and fat distribution.

Furthermore, this study examined the relationship of TBS with multiple clinical variables. Compared to other clinical indicators, HDL-C and TBS were found to be more relevant. Considering that the crowd and r values were small, we further performed a multiple linear regression analysis and observed that the correlation disappeared after adjusting for certain confounding factors. Somehow, HDL-C showed a weak relationship with BMD (β = 0.151, P = 0.034). Since a latest report has determined some effects of HDL-C on bone fragility^32^; and D’Amelio *et al.* also suggested that HDL was significantly higher in osteoporotic patients than controls, the level of HDL could be used as screening for postmenopausal osteoporosis^33^. However, the exact relationship requires further studies. In a previous study, glucose was found to be necessary for Runx2 accumulation, osteoblast differentiation, collagen synthesis, and bone formation^34^. However, there was no correlation between HbA1c% and TBS in our study; this might be due to the inclusion of healthy subjects and relatively small sample size.

There were certain limitations in the current study: small population, lack of more comprehensive clinical information (such as physical activity and dietary habits), not much information on the mechanisms underlying the impact of fat on bone microstructure; all these shortcomings merit further study.

In conclusion, our study demonstrated that body fat mass, especially android fat and visceral fat mass, have negative effects on bone microstructure; while body fat mass contributes to BMD merely through mechanical loading.

## Additional Information

**How to cite this article**: Lv, S. *et al.* Assessment of Fat distribution and Bone quality with Trabecular Bone Score (TBS) in Healthy Chinese Men. *Sci. Rep.* **6**, 24935; doi: 10.1038/srep24935 (2016).

## Figures and Tables

**Table 1:** Anthropometrics, clinical parameters, bone measures, and the correlation with trabecular bone score (TBS) in all 228 Chinese men.

Characteristic	N	Value^#^	Correlation with TBS (r)	P
TBS	228	1.300 ± 0.089	—	—
Age (year)	228	58.780 ± 12.420	−0.142	0.032
Height (cm)	228	171.800 ± 6.614	0.025	0.715
Weight (kg)	228	74.820 ± 10.800	−0.144	0.031
BMI (kg/m^2^)	228	25.460 ± 4.538	−0.115	0.085
Waist circumference (cm)	187	93.000 ± 8.650	−0.173	0.018
Hip circumference (cm)	187	98.770 ± 6.185	−0.031	0.678
FBS (mmol/L)	228	5.405 ± 0.703	0.074	0.280
HbA1c (%)	228	5.776 ± 0.468	0.017	0.796
TG (mmol/L)	228	1.947 ± 1.738	−0.068	0.308
TC (mmol/L)	228	4.789 ± 1.021	0.082	0.218
HDL-C (mmol/L)	228	1.143 ± 0.264	0.161	0.015
LDL-C (mmol/L)	228	3.064 ± 0.776	0.094	0.143
25(OH)VD (ng/ml)	147	45.568 ± 19.016	0.039	0.643
Bone mineral density				
Lumbar Spine (g/cm^2^)	228	1.015 ± 0.162	0.476	<0.001
Hip (g/cm^2^)	228	0.730 ± 0.166	0.339	<0.001
Fat mass (kg)				
Total	209	20.422 ± 4.619	−0.220	0.001
Trunk	209	11.417 ± 2.960	−0.217	0.002
Android	209	2.040 ± 0.618	−0.290	<0.001
Gynoid	209	2.821 ± 0.606	−0.181	0.009
Limbs	209	7.797 ± 2.246	−0.235	<0.001
Lean mass (kg)				
Total	209	52.893 ± 8.526	0.037	0.597
Trunk	209	26.612 ± 6.109	−0.097	0.162
Android	209	3.933 ± 0.704	−0.121	0.081
Gynoid	209	7.694 ± 1.374	0.030	0.670
Limbs	209	22.712 ± 4.242	−0.021	0.767
Subcutaneous fat (g)	95	171.554 ± 49.291	−0.079	0.449
Visceral fat (g)	95	147.609 ± 44.518	−0.271	0.008

^#^Values shown in Mean ± S.D.

**Table 2:** Multiple linear regression analyses of TBS and BMD on fat distribution.

Variables	Standardized β	t	P
TBS			
Age	−0.212	−2.879	0.004
BMI	0.038	0.382	0.703
Android fat mass	−0.611	−3.559	<0.001
Gynoid fat mass	0.166	1.217	0.225
Trunk fat mass	0.208	1.097	0.274
Limbs fat mass	−0.110	−1.151	0.251
HDL-C	0.099	1.143	0.154
HbA1c%	0.137	1.912	0.057
Lumbar spine BMD			
Age	−0.062	−0.824	0.411
BMI	0.101	0.999	0.319
Android fat mass	−0.120	−0.679	0.498
Gynoid fat mass	0.220	1.575	0.117
Trunk fat mass	0.198	1.019	0.310
Limbs fat mass	−0.100	−1.023	0.308
HDL-C	0.151	2.131	0.034
HbA1c%	0.139	1.888	0.061

Model was adjusted for age, BMI, HDL-C and HbA1c%.

TBS: trabecular bone score, BMD: bone mineral density, BMI: body mass index.

**Table 3:** Multiple linear regression analyses of TBS and BMD on subcutaneous and visceral fat mass.

Variables	Standardized β	t	P
TBS			
Age	0.126	1.084	0.281
BMI	0.150	0.998	0.321
Subcutaneous fat mass	−0.077	−0.611	0.543
Visceral fat mass	−0.280	−2.422	0.017
Lumbar spine BMD			
Age	0.327	2.904	0.005
BMI	0.313	2.157	0.034
Subcutaneous fat mass	0.137	0.130	0.262
Visceral fat mass	−0.075	−0.667	0.506

Model was adjusted for age and BMI.

TBS: trabecular bone score, BMD: bone mineral density, BMI: body mass index.
